# High-resolution structures of *Lactobacillus salivarius* transketolase in the presence and absence of thiamine pyrophosphate

**DOI:** 10.1107/S2053230X1501657X

**Published:** 2015-09-23

**Authors:** Petra Lukacik, Carina M. C. Lobley, Mario Bumann, Victoria Arena de Souza, Raymond J. Owens, Paul W. O’Toole, Martin A. Walsh

**Affiliations:** a Diamond Light Source, Harwell Science and Innovation Campus, Didcot OX11 0DE, England; b Research Complex at Harwell, R92 Rutherford Appleton Laboratories, Harwell OX11 0FA, England; c MRC France, BM14, c/o ESRF, 6 Rue Jules Horowitz, BP 220, 38043 Grenoble, France; d Oxford Protein Production Facility UK, Research Complex at Harwell, R92 Rutherford Appleton Laboratories, Harwell OX11 0FA, England; e Department of Microbiology, Alimentary Pharmabiotic Centre, University College Cork, Cork, Ireland

**Keywords:** transketolase, *Lactobacillus salivarius*

## Abstract

Probiotic bacterial strains have been shown to enhance the health of the host through a range of mechanisms including colonization, resistance against pathogens, secretion of antimicrobial compounds and modulation of the activity of the innate immune system. *Lactobacillus salivarius* UCC118 is a well characterized probiotic strain which survives intestinal transit and has many desirable host-interaction properties. Probiotic bacteria display a wide range of catabolic activities, which determine their competitiveness *in vivo*. Some lactobacilli are heterofermentative and can metabolize pentoses, using a pathway in which transketolase and transaldolase are key enzymes. *L. salivarius* UCC118 is capable of pentose utilization because it encodes the key enzymes on a megaplasmid. The crystal structures of the megaplasmid-encoded transketolase with and without the enzyme cofactor thiamine pyrophosphate have been determined. Comparisons with other known transketolase structures reveal a high degree of structural conservation in both the catalytic site and the overall conformation. This work extends structural knowledge of the transketolases to the industrially and commercially important *Lactobacillus* genus.

## Abstract 2

The crystal structure of *L. salivarius* transketolase has been determined to high resolution in the presence and absence of thiamine pyrophosphate. The structures are presented with a brief comparison with other known transketolase structures.

## 1. Introduction   

The Gram-positive, lactic acid bacterium *Lactobacillus salivarius* UCC118 (Claesson *et al.*, 2006 ▸) is of particular interest as a member of a group of probiotic bacteria (Neville & O’Toole, 2010 ▸) that successfully colonize the human gastro-intestinal tract, conferring such health benefits as prevention or hindrance of intestinal infection, elimination of food-borne pathogens (Corr *et al.*, 2007 ▸) and reduction in inflammation and food intolerance (Sheil *et al.*, 2004 ▸). Most lactobacilli that are considered probiotic have undergone reductive genome evolution to dispense with the metabolic pathways used for substrate catabolism in the environment outside the host (Makarova *et al.*, 2006 ▸). *L. salivarius* UCC118 is unusual because it retains the genes for pentose utilization by harbouring some of them on the chromosome and some of them on a 242 kb pMP118 megaplasmid (Claesson *et al.*, 2006 ▸). More specifically, megaplasmid-encoded transaldolase (LSL_1888, *mipB*) and transketolase (LSL_1946, *tktA*) complete the pentose phosphate pathway of *L. salivarius* UCC118. This is predicted to give it a competitive advantage when ribose, abundant in plant material, is present in the diet.

The generic transketolase (EC 2.2.1.1; Tkt) is a ubiquitous enzyme that catalyses the cleavage of a carbon–carbon bond adjacent to a carbonyl group of a ketose sugar and transfers a two-carbon moiety to an aldose sugar. There are a variety of donor and acceptor sugar phosphates that can be converted by transketolase, making it, along with the enzyme transaldolase, a central enzyme in the link between the pentose phosphate pathway and glycolysis (Lindqvist *et al.*, 1992 ▸). In photosynthetic organisms Tkt also catalyses reactions in the Calvin cycle. A cofactor, thiamine pyrophosphate (TPP), and a divalent metal ion, Mg^2+^ by preference, are required for catalysis. In *Saccharomyces cerevisiae* and other species, Ca^2+^, Mn^2+^ and Co^2+^ can all replace the Mg^2+^ ion and retain catalytic function (Lindqvist *et al.*, 1992 ▸). In addition to its significant metabolic role, Tkt may also be relevant as a catalyst for the industrial organic synthesis of pure chiral products (Nikkola *et al.*, 1994 ▸). Both its broad substrate specificity and its ability to catalyse the formation of asymmetric C—C bonds make the Tkt enzyme an attractive biocatalyst.

Several Tkt crystal structures have been published, with a particular emphasis on the *S. cerevisiae* enzyme (for a comprehensive review, see Schneider & Lindqvist, 1998 ▸). Structures have been determined of *S. cerevisiae* Tkt in the absence of TPP or metal ions (Sundström *et al.*, 1992 ▸), in the presence of TPP and a Ca^2+^ ion (Lindqvist *et al.*, 1992 ▸) and in complex with thiamine thiazolone diphosphate, a reaction-intermediate analogue (Nilsson *et al.*, 1993 ▸), as well as with three TPP analogues to further investigate binding (König *et al.*, 1994 ▸). Taken together, these studies provide a considerable understanding of the mode of cofactor and divalent metal-ion binding and a good understanding of the enzyme mechanism. More recently, the structure of human transketolase has been solved, providing the first example of the mammalian enzyme (Mitschke *et al.*, 2010 ▸). While the human transketolase structure is similar to those of the yeast and bacterial enzymes (2.1 Å r.m.s. deviation on superposition with the *S. cerevisiae* structure), the tighter substrate specificity in the human enzyme has been explained by a narrower substrate channel. In this work, we present high-resolution crystal structures of the *L. salivarius* UCC118 Tkt protein (*Ls*TktA) in the presence and absence of the cofactor TPP and Mg^2+^ ions, which have been determined as part of a directed structural genomics approach to furthering our understanding of how these bacterial strains colonize and persist in the human gut and enhance the wellbeing of the host.

## 2. Materials and methods   

### 2.1. Protein production and crystallization   

Using the In-Fusion method, the coding sequence for *L. salivarius tktA* was cloned into the pOPINF plasmid (Berrow *et al.*, 2007 ▸). The protein was produced in *Escherichia coli* strain Rosetta pLysS (DE3) using auto-induction with TB Overnight Express medium (Novagen). The cells were harvested by centrifugation and stored at −80°C. The cells were lysed and the soluble fraction was then purified *via* nickel-chelation chromatography and subsequent gel-filtration chromatography. Fractions containing protein were identified by SDS–PAGE and pooled. The purified protein was concentrated to 12 mg ml^−1^ for crystallization.

In order to co-crystallize *Ls*TktA with its cofactor, 200 m*M* stocks of MgCl_2_ and TPP (Sigma) were prepared in distilled water and 1 *M *Tris pH 7.5 buffer, respectively. The stocks were added directly to the concentrated protein to a final concentration of 2 m*M* MgCl_2_ and 0.5 m*M* TPP. Screening of *Ls*TktA co-crystallization conditions were carried out as published elsewhere (Walter *et al.*, 2005 ▸). Initial crystal hits were optimized by the hanging-drop vapour-diffusion method in EasyXtal 15-well plates (Qiagen) at 293 K. The best crystals were obtained by mixing 1 µl of the protein concentrated to 12 mg ml^−1^ with 1 µl reservoir solution consisting of 15%(*w*/*v*) PEG 3350, 0.1 *M* sodium acetate, 0.1 *M* bis-tris propane pH 7.5 (Hampton Research).

Apo crystals were similarly grown in a hanging-drop setup by mixing 1 µl *Ls*TktA (12 mg ml^−1^) with 1 µl 20%(*w*/*v*) PEG 3350, 0.2 *M* NaCl essentially as described by Horsham *et al.* (2010 ▸).

Cryoprotection of both the co-crystals and the apo crystals was achieved by rapidly transferring the crystals from their mother liquor into a droplet of the crystal reservoir solution supplemented with 20%(*v*/*v*) ethylene glycol and then immediately flash-cooling them in liquid nitrogen. Diffraction from these crystals was inconsistent even amongst crystals mounted from the same drop. Consequently, a substantial number of crystals had to be screened prior to obtaining atomic resolution diffraction. X-ray diffraction data were ultimately collected at 100 K on beamline I04-1 at Diamond Light Source (DLS), Didcot, England using a Pilatus 2M detector. Data were processed using the *xia*2 automated data-reduction pipeline (Winter, 2010 ▸), which makes use of *MOSFLM* (Leslie, 2006 ▸), *POINTLESS* (Evans, 2006 ▸), *CCP*4 (Winn *et al.*, 2011 ▸) and *XDS* (Kabsch, 2010 ▸).

### 2.2. X-ray data collection and structure determination   

The crystal structure of thiamine pyrophosphate-bound Tkt was solved by molecular replacement using *MrBUMP* (Keegan & Winn, 2007 ▸; Murzin *et al.*, 1995 ▸; Pearson & Lipman, 1988 ▸) with the protein sequence of Tkt from *L. salivarius* UCC118 (UniProt Q1WQU8). Using a model prepared by *CHAINSAW* (Stein, 2008 ▸) based on chain *B* of the TPP-bound *Bacillus anthracis* Tkt structure (PDB entry 3m49; Center for Structural Genomics of Infectious Diseases, unpublished work), *MrBUMP* was able to provide a molecular-replacement solution with *MOLREP* (Vagin & Teplyakov, 2010 ▸) with an *R*_free_ of 0.48. The structure was manually rebuilt with iterative rounds of rebuilding in *Coot* (Emsley & Cowtan, 2004 ▸) and refinement with *REFMAC*5 (Murshudov *et al.*, 2011 ▸). In the early stages, refinement was carried out with a low weighting term and isotropic *B* factors before relaxing the refinement parameters at the later stages of rebuilding. The quality of the final model was assessed using *PROCHECK* (Laskowski *et al.*, 1993 ▸), the *RCSB Validation Server* (Berman *et al.*, 2000 ▸, 2003 ▸) and *MolProbity* (Chen *et al.*, 2010 ▸; Lovell *et al.*, 2003 ▸). The statistics associated with the final model are detailed in Table 1 ▸.

### 2.3. Size-exclusion chromatography and multi-angle laser light scattering (SEC-MALLS)   

The molar mass (MW) and MW distributions of monomeric and dimeric Tkt species were determined on a ÄKTA pure chromatography system equipped with a Superdex 200 Increase 10/300 GL column (catalogue No. 28-9909-44). The sample was applied onto the column at a flow rate of 0.7 ml min^−1^ in a buffer consisting of 20 m*M* Tris–HCl pH 7.5, 200 m*M* NaCl, 0.5 m*M* TCEP (20°C) with and without the addition of 0.5 m*M* TPP. The MALLS system was a Wyatt DAWN HELEOS II with an added WyattQELS dynamic light-scattering unit connected to a Wyatt Optilab T-rEX refractive-index detector. The data were analysed using the Wyatt *ASTRA* 6 software.

### 2.4. Amino-acid sequence analysis   

Amino-acid sequence alignment and phylogenetic tree generation was carried out using *ClustalW*2 (Thompson *et al.*, 1994 ▸). The tree was visualized using the online application *iTOL* (Letunic & Bork, 2006 ▸).

## 3. Results and discussion   

### 3.1. Crystal and solution structure of transketolase from *L. salivarius* UCC118   

**Figure 1:**
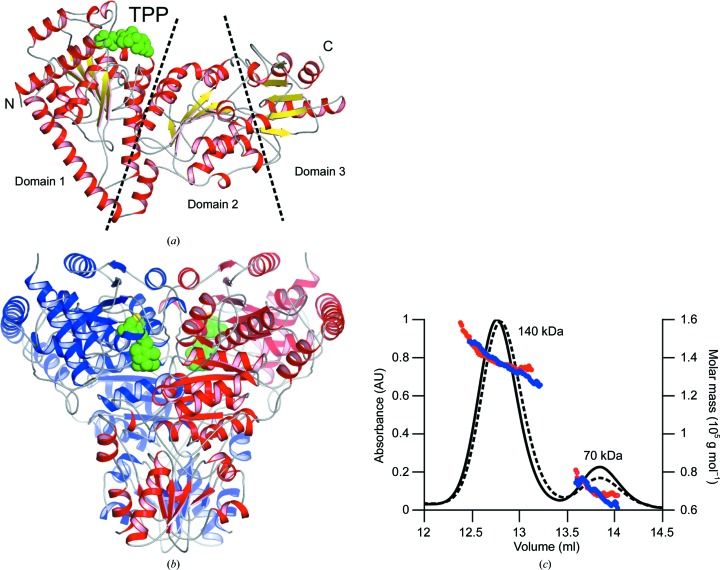
(*a*) The crystal structure of the *L. salivarius* Tkt protomer has a V-shaped conformation that can be divided into three discrete domains. Secondary-structure elements are coloured yellow for β-strands and red for α-helices, with loop regions in grey. The TPP ligand molecule is shown in spherical representation and is coloured green. (*b*) Two Tkt protomers coloured red and blue associate through an extensive interface to form a functional dimer. Molecular-graphics figures were prepared using *PyMOL* (v.1.3r1; Schrödinger). (*c*) SEC-MALLS analysis of *Ls*Tkt. The continuous black line shows the normalized protein absorbance (arbitrary units) as a function of elution volume for the apoprotein and the dashed black line is that for *Ls*TktA in the presence of 0.5 m*M* TPP. The red and blue scatter plots represent the molar-mass distributions of the presumed dimeric and monomeric species of the apoprotein and cofactor-bound protein, respectively.

*Ls*TktA crystallizes in the trigonal space group *P*3_2_21 with a protomer in the asymmetric unit. The structure was solved by molecular replacement using the *B. anthracis* Tkt structure (PDB entry 3m49), which has 56% sequence identity, as a model. The final electron-density maps allowed the modelling of the majority of the polypeptide chain and resulted in a model consisting of 662 amino acids along with 246 water molecules in the apo structure and 225 water molecules in the TPP-bound structure. The *Ls*TktA monomer adopts the expected overall V-shaped transketolase fold consisting of three α/β domains (Fig. 1 ▸ *a*). The *Ls*TktA dimer was generated by crystal symmetry and was validated using *PISA* (http://www.ebi.ac.uk/pdbe/prot_int/pistart.html; Krissinel & Henrick, 2007 ▸; ). This homodimer forms two enzyme active sites at its broad interface, with residues from both monomers contributing to each active site (Fig. 1 ▸ *b*). Overall, apo-monomer dimerization buries an interface area of 3998 Å^2^ of each subunit with the formation of 16 salt bridges, 60 hydrogen bonds and numerous hydrophobic interactions predominantly involving residues from the first two domains of each monomer (residues 1–528).

The presence of the *Ls*TktA homodimer in solution was confirmed by size-exclusion chromatography and multi-angle laser light scattering (SEC-MALLS; Fig. 1 ▸ *c*). Two peaks corresponding to molar masses (MWs) of 138.8 (±0.8) and 70.4 (±2.4) kDa were observed for the apo protein, corresponding closely to the theoretical dimeric and monomeric MWs calculated from the amino-acid sequence of the histidine-tagged Tkt, which consists of 680 amino acids. The peak distribution indicated approximately 75% dimeric and 25% monomeric species in solution. These observations correlate well with analytical centrifugation experiments that have shown Tkt from *S. cerevisiae* to be a dimer which dissociates at low concentrations (<0.1 mg ml^−1^) in the absence of the coenzyme (Cavalieri *et al.*, 1975 ▸). Our experiments were carried out in a similar concentration range (1 mg ml^−1^) in the absence and presence of TPP.

### 3.2. *Ls*TktA active site and comparative analysis with other transketolases   

For the last two decades considerable research into deciphering the catalytic mechanism of Tkt has been made and includes comprehensive studies of the enzymes from *S. cerevisiae* and *E. coli*. This body of work provides significant understanding of the catalytic mechanism and has provided insights into the functionality of several invariant residues. For a comprehensive list of the key invariant residues and their potential function, see Nikkola *et al.* (1994 ▸). Here, we will discuss the subtle differences between the active sites of *Ls*TktA and the *S. cerevisiae* and human transketolases.

**Figure 2:**
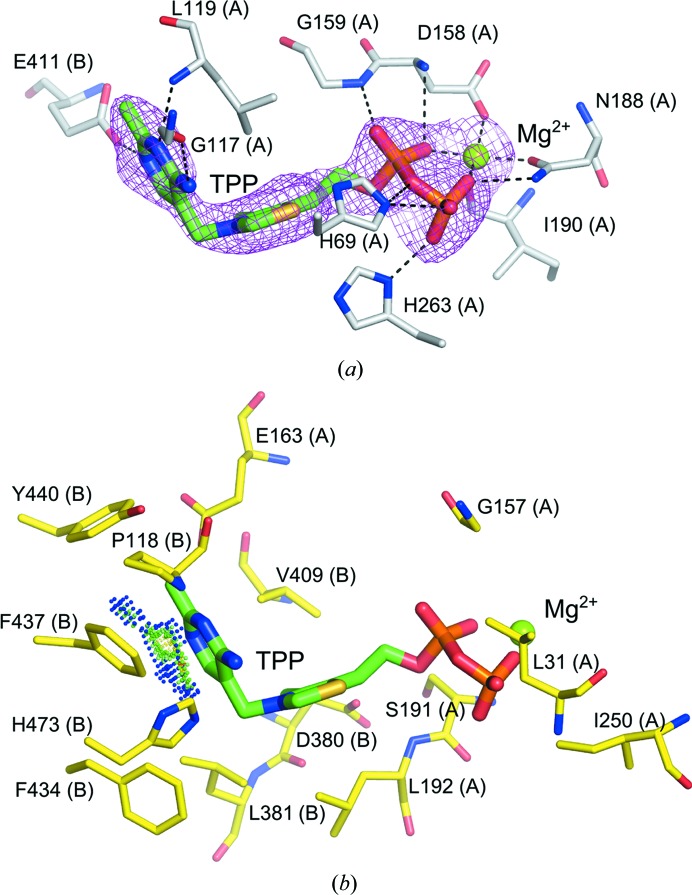
The cofactor site of *Ls*TktA. (*a*) The TPP cofactor is shown in stick representation with C atoms coloured green and the Mg^2+^ ion is represented as a light green sphere. The magenta mesh represents the *F*_o_ − *F*_c_ map contoured at 3σ around the metal and cofactor. Residues interacting with TPP/Mg^2+^ through hydrogen bonding (dashed lines) are shown in stick representation with the C atoms coloured grey. A distance cutoff range of 1.9–3.3 Å was used for hydrogen bonds in all figures. (*b*) Residues contributing hydrophobic interactions are shown as yellow sticks. Small blue and green spheres represent the favourable π-­stacking between Phe437 and the TPP aminopyridine (van der Waals contacts generated using *PROBE*; Word *et al.*, 2000 ▸). Interacting residues were identified using the program *LigPlot*+ (Wallace *et al.*, 1995 ▸).

Two symmetrical TPP cofactor-binding sites are located at the *Ls*TktA dimer interface. The N-terminal α/β domain of one monomer is responsible for TPP cofactor pyrophosphate binding through a number of hydrogen bonds (Fig. 2 ▸ *a*). The central domain of the second monomer interacts with the aminopyridine portion through hydrophobic interactions, including π-stacking with Phe437 (Fig. 2 ▸ *b*). The C2 atom of the TPP thiazolium ring is solvent-exposed *via* a tunnel wide enough for the sugar substrate. The thiazolium ring is believed to act as an electron sink, in part stabilizing the α-carbanion intermediate formed during catalysis (Fiedler *et al.*, 2002 ▸). Such constrained access to the catalytic site is consistent with the bi-bi ping-pong mechanism proposed for this enzyme (Nilsson *et al.*, 1997 ▸). The *Ls*TktA structure also contains an Mg^2+^ ion coordinated jointly by the protein and the TPP pyrophosphate group. The transketolases require a divalent metal ion, preferably Mg^2+^, for catalytic activity.

**Figure 3:**
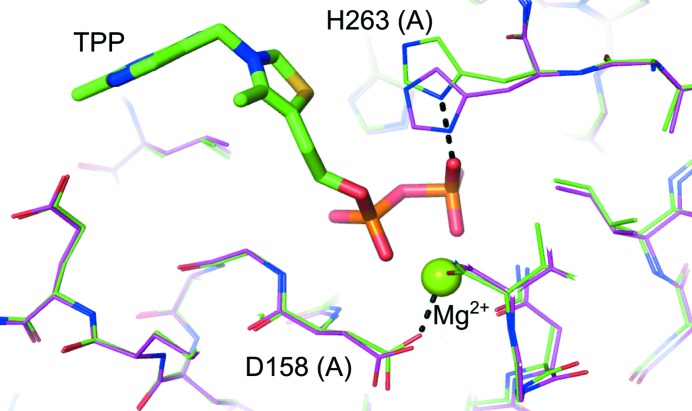
Superimposition of the apo and TPP-bound *Ls*TktA structures: His263 adopts an altered side-chain conformation in the cofactor-bound structure (green) relative to the apoprotein (magenta).

TPP-induced perturbations are restricted to very fine rearrangements of the active site; the apo and TPP-bound structures of Tkt are globally highly similar, with an average C^α^ r.m.s.d. of 0.29 Å as determined by the *CCP*4 program *LSQKAB* (Winn *et al.*, 2011 ▸). His263 adopts an altered side-chain conformation that allows it to make a hydrogen bond to the TPP terminal phosphate (Fig. 3 ▸) and Asp158 has an adjusted side-chain position that enables it to coordinate to the Mg^2+^ ion. The histidine residue equivalent to His263 in the *S. cerevisiae* enzyme is responsible for interacting with the TPP pyrophosphate as well as for substrate recognition and binding (Nilsson *et al.*, 1997 ▸; Wikner *et al.*, 1997 ▸). The conformational flexibility observed in *Ls*TktA is likely to be needed to accommodate both roles. Other changes involve small shifts in the loop 190–198, which is clearly resolved in both structures. This differs from the structure of *S. cerevisiae* Tkt, where two flexible loops (region 185–198 and 383–393) could not be modelled owing to an absence of electron density in the apo structure. On binding of the cofactor these regions become clear in the electron-density map and consequently are believed to be rigidified by the binding of the cofactor (Sundström *et al.*, 1992 ▸).

**Figure 4:**
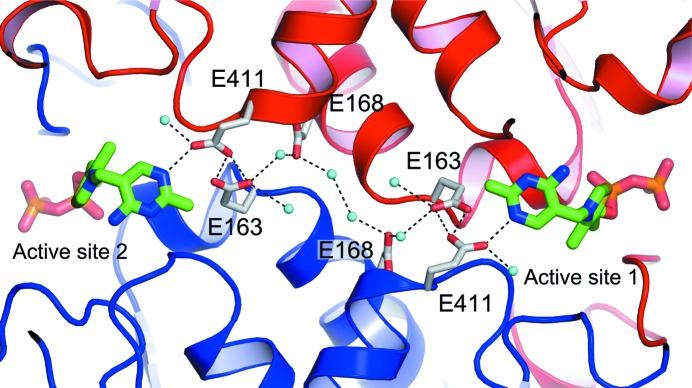
A network of glutamate residues and water molecules connects the two active sites of the Tkt dimer. The chains contributed by the two protomers are coloured red and blue. Hydrogen bonds are marked in dashed lines and water molecules are represented by light blue spheres.

The majority of the active-site residues are identical between *L. salivarius* and *S. cerevisiae*. These include (i) the residues interacting with the pyrophosphate of TPP, which include two conserved histidines (His69, His263, Asp158, Gly159 and Asn188), (ii) the residues responsible for metal binding (Asp158, Asn188 and Ile190), (iii) the residues involved in substrate binding and recognition (His28, His263, Ser384, Arg357, Arg520, His461 and Asp469) and (iv) the residues that interact with the TPP pyrimidine ring (Gly117, His473, Leu119, Glu411, Phe434, Phe437 and Tyr440) (Fig. 2 ▸). The latter interactions include a conserved glutamate, Gly411, which forms a hydrogen bond to the N1 atom of the pyrimidine ring. The protonation state of Glu411 affects the electronic properties of the pyrimidine amino group, which can then influence the state of the C2 atom of the thiazolium ring, promoting catalytic activity (Lindqvist *et al.*, 1992 ▸). The side chain of Glu411 is stabilized by interactions with the side chain of Glu163, which is connected to Glu168 through a structural water molecule. In turn, Glu168 is linked through two structural waters to the symmetry-related Glu168, Glu163 and Glu411 and to the N1 atom of the pyrimidine ring in the second active site (Fig. 4 ▸). This network of interactions that is observed in the *L. salivarius* Tkt protein has previously been proposed to provide a mechanism by which the active site can ‘sense’ the cofactor occupancy in the second site (Nikkola *et al.*, 1994 ▸).

It is only in the interactions with the thiazole ring (Leu192, Asp380, Leu381 and Val409) that some conservative substitutions are tolerated. Specifically, Leu192 and Val409 replace the *S. cerevisiae* Ile191 and Ile416, respectively. However, the same substitutions are also observed in sequences from other organisms, including the Gram-positive bacterium *B. anthracis* and the epsilonproteobacterium *Campylobacter jejuni*.

A comparison between the active sites of *Ls*TktA and human Tkt also reveals a high degree of homology. However, there are certain key differences located near the active site. Firstly, the conserved His473, which interacts with the TPP pyrimidine ring in *Ls*TktA, is replaced by a glutamine in mammalian Tkts. This substitution is very conservative since it retains an amino moiety to interact with the pyrimidine ring. Secondly, Leu192 in *Ls*TktA superposes with Gln189 in human Tkt. This residue is positioned at the edge of the thiazole ring-binding pocket and the presence of glutamine in the human enzyme is believed to hinder cofactor release after catalysis (Mitschke *et al.*, 2010 ▸). Finally, Ser265 in *Ls*TktA is substituted by a lysine in the human structure. This lysine is likely to narrow the substrate-binding pocket, explaining the different substrate specificities between the human and the bacterial Tkt enzymes (Mitschke *et al.*, 2010 ▸).

### 3.3. Global comparison of known Tkt structures   

**Figure 5:**
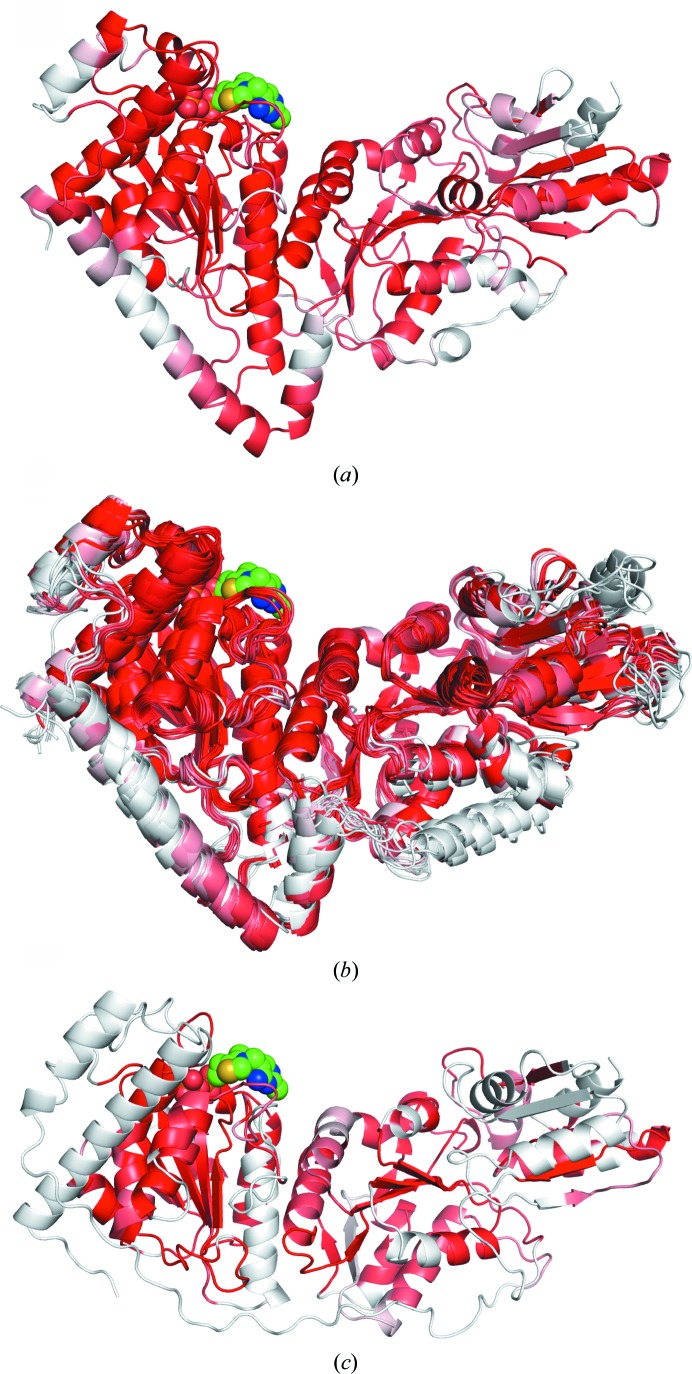
Analysis of global conformation changes between transketolases generated by *ProSMART* and visualized in *PyMOL*. The residues are coloured according to the similarity of their local coordinate frames. Residues that relate closely to the rigid substructure are coloured red, gradually fading to white for regions that adopt a different global conformation. (*a*) *Ls*TktA (PDB entry 4c7x), (*b*) superimposition of nonhuman transketolases (PDB entries 1ay0, 1r9j, 3kom, 3upt, 1itz, 2e6k, 3m7i, 1qgd, 3hyl and 3rim) and (*c*) human transketolase (PDB entry 3mos).

Tkt has been studied from a number of organisms, and coordinates are available for apo structures from *Saccharo­myces cerevisiae* (Lindqvist *et al.*, 1992 ▸), *Francisella tularensis* (PDB entry 3kom), *Bacillus anthracis* (PDB entry 3hyl), *Campylobacter jejuni* (PDB entry 3l84) and *Thermus thermophilus* (PDB entry 2e6k) and for a selection of cofactor-bound, substrate-bound and analogue-bound Tkt structures from *S. cerevisiae* (PDB entries 1gpu, 1ay0, 1tka, 1tkb and 1tkc; Fiedler *et al.*, 2002 ▸; König *et al.*, 1994 ▸; Wikner *et al.*, 1997 ▸), *Escherichia coli* (PDB entries 1qgd, 2r8o, 2r8p and 2r5n; Littlechild *et al.*, 1995 ▸), *Leishmania mexicana* (PDB entry 1r9j; Veitch *et al.*, 2004 ▸), *B. anthracis* (PDB entry 3m49), *C. jejuni* (PDB entries 3m6l, 3m34 and 3m7i), *Homo sapiens* (PDB entries 3mos and 3ooy; Mitschke *et al.*, 2010 ▸), *Burkholderia thailandensis* (PDB entry 3uk1), *B. pseudomallei* (PDB entry 3upt), *Mycobacterium tuberculosis* (PDB entry 3rim; Fullam *et al.*, 2012 ▸) and *Zea mays* (PDB entry 1itz; Gerhardt *et al.*, 2003 ▸). Structures that lack citations have been submitted directly to the PDB as part of various structural genomics initiatives. These structures were compared using *ProSMART*, a tool that produces conformation-independent structural comparisons of structures based on the conservation of local structure (Nicholls, 2011 ▸). Each chain from each structure has been superposed on apo *L. salivarius* Tkt and the TPP-bound enzyme. In comparison to the apo structure, 48 of the 54 superpositions had a global r.m.s.d. of less than 2 Å, with the largest r.m.s.d.s (∼7 Å) observed for the human Tkt structures. Comparison with the TPP-bound structure gave 50 of 54 superpositions with an r.m.s.d. of less than 2 Å, again with the largest outliers (∼8 Å) being human Tkt. Colour visualization of global structural differences using *ProSMART* shows that *Ls*TktA clusters with nonhuman transketolases (Fig. 5 ▸). This very high structural homology corresponds to a high sequence homology of 40–60% between all of the structures compared, with the exception of human Tkt. The close structural and sequence homology between Tkt from such a wide variety of organisms reflects the central role that the enzyme has in metabolism and the conservation of protein sequence, structure and catalytic functionality across several kingdoms of life. Human Tkt has only 25–26% sequence identity to *LsTktA*. This is reflected in the significantly reduced structural homology between *L. salivarius* and human Tkt, which is presumably a consequence of the significant evolutionary distance between the two species (Figs. 5 ▸ and 6 ▸).

## 4. Conclusions   

The production of many commercially important foodstuffs is reliant on lactobacilli. Besides a role in human nutrition, lactobacilli also widely colonize the human gastrointestinal and genitourinary tracts, and some species have been attributed with conferring diverse health benefits on their host (Lebeer *et al.*, 2008 ▸). In the case of *L. salivarius* UCC118 some of these positive ‘probiotic’ effects are illustrated by its role in alleviating certain symptoms of irritable bowel syndrome (Ortiz-Lucas *et al.*, 2013 ▸). Potential health and commercial motivations for probiotic exploitation have raised interest in the genetic characterization of different *Lactobacillus* species. This in turn has identified extragenomic plasmids as important for the fitness, stress resistance, competitiveness and metabolic expansion of these bacteria (Li *et al.*, 2007 ▸). In this work, we have attempted to go beyond genetic characterization of the *L. salivarius* pMP118 megaplasmid and towards its structural annotation. With this aim, we have solved crystal structures of Tkt from *L. salivarius* UCC118 in the presence and absence of the cofactor TPP and the catalytic Mg^2+^ ion. The two structures are globally very similar, with some slight side-chain rearrangements triggered by cofactor binding. Comparison of the two novel structures presented here with those previously determined for transketolases with coordinates deposited in the PDB reveals strong sequence and structure homology between species. Broadly, the global r.m.s.d.s observed are within the error of the experiment, although there are larger deviations from the less closely related human enzyme. This work adds to the extensive body of information accumulated about the structure of Tkt from a variety of organisms and extends the structural knowledge to the industrially important *Lactobacillus* genus.

Whilst the structural differences between the tranketolase family members may appear to be minor, understanding of these fine differences may prove to be exploitable for the modification of the bacterium for more efficient pentose utilization or for the selection and engineering of a maximally efficient and commercially viable transketolase biocatalyst. A particularly attractive area of application is the modification of microorganisms for efficient biofuel production, where high substrate utilization and metabolic fluxes are required (Alper & Stephanopoulos, 2009 ▸).

## Supplementary Material

PDB reference: transketolase, apo, 4c7v

PDB reference: cofactor-bound, 4c7x

## Figures and Tables

**Figure 6:**
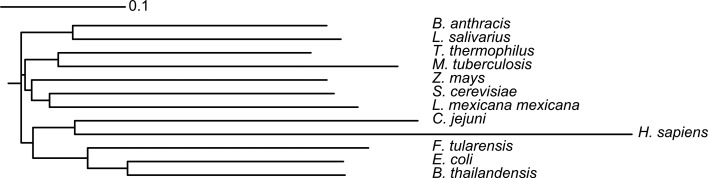
Phylogenetic tree of Tkt amino-acid sequences for which structures are available in the PDB. The lengths of the branches indicate the number of substitutions as a proportion of the length of the alignment (excluding gaps).

**Table 1:** *Ls*TktA data-collection and refinement statistics Values in parentheses are for the outermost resolution shell.

	Apo *Ls*TktA	TPP-bound *Ls*TktA
Data collection		
X-ray source	I04-1, DLS	I04-1, DLS
Wavelength ()	0.917	0.917
Space group	*P*3_2_21	*P*3_2_21
Unit-cell parameters (, )	*a* = *b* = 76.15, *c* = 194.51, = = 90, = 120	*a* = *b* = 74.99, *c* = 192.85, = = 90, = 120
Resolution ()	39.142.20 (2.262.20)	53.872.29 (2.352.29)
*R*_merge_ †	0.064 (0.624)	0.122 (0.685)
Mosaicity ()	0.08	0.34
*I*/(*I*)	17.8 (2.8)	15.0 (3.5)
Completeness (%)	99.7 (99.9)	100.0 (100.0)
Multiplicity	6.6 (6.8)	9.7 (10.2)
Refinement		
No. of reflections	223690 (16857)	283055 (21815)
No. of unique reflections	33992 (2488)	29191 (2138)
*R*_cryst_ ‡	0.162 (0.238)	0.154 (0.181)
*R*_free_ ‡	0.219 (0.273)	0.236 (0.291)
No. of non-H atoms		
Protein	5092	5092
Ligands	0	27
Water	246	225
Average *B* factors (^2^)		
Protein	44.5	33.9
TPP	N/A	43.7
Mg^2+^	N/A	31.9
Waters	41.2	31.9
R.m.s. deviations		
Bond lengths ()	0.017	0.018
Bond angles ()	1.780	1.857
Ramachandran statistics (%)		
Favoured regions	96.5	96.5
Additionally allowed	2.9	3.35
Outliers	0.6	0.15
*MolProbity* clashscore	3.65 [99th percentile]	3.63 [99th percentile]
PDB code	4c7v	4c7x

† *R*_merge_ = 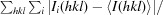 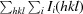.

‡ *R*_cryst_ = 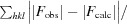 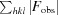, where *F*_obs_ and *F*_calc_ are the observed and calculated structure-factor amplitudes, respectively. *R*_free_ is calculated as for *R*_cryst_ but using a random 5% subset of the data that were excluded from the refinement.
